# Multigeneration Inheritance through Fertile XX Carriers of an *NR0B1* (*DAX1*) Locus Duplication in a Kindred of Females with Isolated XY Gonadal Dysgenesis

**DOI:** 10.1155/2012/504904

**Published:** 2012-02-28

**Authors:** Michela Barbaro, Jackie Cook, Kristina Lagerstedt-Robinson, Anna Wedell

**Affiliations:** ^1^Department of Molecular Medicine and Surgery, Karolinska Institut, Karolinska University Hospital, CMM L8:02, 17176 Stockholm, Sweden; ^2^Department of Clinical Genetics, Sheffield Children's Hospital, Sheffield S 102 TH, UK

## Abstract

A 160 kb minimal common region in Xp21 has been determined as the cause of XY gonadal dysgenesis, if duplicated. The region contains the *MAGEB* genes and the *NR0B1* gene; this is the candidate for gonadal dysgenesis if overexpressed. Most patients present gonadal dysgenesis within a more complex phenotype. However, few independent cases have recently been described presenting with isolated XY gonadal dysgenesis caused by relatively small *NR0B1* locus duplications. We have identified another *NR0B1* duplication in two sisters with isolated XY gonadal dysgenesis with an X-linked inheritance pattern. We performed X-inactivation studies in three fertile female carriers of three different small *NR0B1* locus duplications identified by our group. The carrier mothers did not show obvious skewing of X-chromosome inactivation, suggesting that *NR0B1* overexpression does not impair ovarian function. We furthermore emphasize the importance to investigate the *NR0B1* locus also in patients with isolated XY gonadal dysgenesis.

## 1. Introduction

Xp21 duplications containing the *NR0B1* (*DAX1*) locus have long been known to be associated with XY gonadal dysgenesis (GD). A 160-kb minimal common region that, if duplicated, causes XY GD has also been determined [1] by comparing several patients with Xp rearrangements. This minimal region contains the *MAGEB* genes and the *NR0B1* (*DAX1*) gene, with *NR0B1 *as the strongest candidate to cause gonadal dysgenesis if overexpressed [1, 2]. In fact *NR0B1* has an embryonic expression compatible with a role in sex determination and in adrenal and hypothalamic function in mice [3], and several functional roles in the hypothalamic-pituitary-adrenal-gonadal axis have been reported [4]. However, XY mice transgenic for *Dax1* show delayed testis development and sex reversal only if the transgene is tested against a weak *Sry* allele [5]. In humans, the effect of *NR0B1* overexpression is also shown by XY patients with 1p duplications including the *WNT4* gene; WNT4 is a signalling molecule that has been shown to upregulate *NR0B1* and XY patients with *WNT4* duplications present with abnormal gonadal development [6]. Another indirect proof in human patients is provided by patients with XY GD caused by *NR5A1 *(*SF1*) haploinsufficiency. *NR0B1* has been reported to inhibit NR5A1, with consequent reduction of steroidogenesis and AMH production, thus it can be hypothesized that overexpression of *NR0B1* leads to gonadal dysgenesis via inhibition of NR5A1.

Although the aforementioned data support *NR0B1* as the gene responsible for GD, a direct proof in a patient is still missing as a duplication containing only the *NR0B1* gene has not been identified yet in XY patients with GD. All duplications reported so far also contain at least some of the *MAGEB* genes that have specific testis expression but yet unknown function; thus, they cannot be ignored.

Most patients reported before the development of array-CGH had XY GD as part of a more complex phenotype which also included dysmorphic features and/or mental retardation (for review [7, 8]). This is explained by the fact that most patients have been identified by conventional karyotyping; thus, they carry large genomic rearrangements involving several genes.

We have previously described two *NR0B1* locus duplications in three patients with isolated XY GD; two sisters with complete XY GD [9] and a boy with partial XY GD [10]. Both duplications were of a relatively small size (≤800 kb). In fact they were not identified by conventional karyotyping but by array-CGH and MLPA, respectively. This indicates that *NR0B1* locus duplications should be investigated also in cases with isolated XY GD and not only when GD, in partial or complete form, is part of a more complex phenotype. In fact two other groups have applied array-CGH to patients with 46,XY GD and have identified additional *NR0B1* locus duplications [11, 12].

We developed a DSD probe set for MLPA analysis to screen for dosage imbalances of several genes involved in DSD, with particular attention to genes involved in GD [10]. This probe set includes probes for *NR0B1 *and also the *MAGEB1* gene, thus enabling us to detect small *NR0B1* locus duplications. Here, we present a *NR0B1* locus duplication responsible for isolated XY GD identified in a large English family. We also analysed the X-inactivation patterns in fertile female carriers of each of the three small *NR0B1* locus duplications identified by our group. Female carriers of macroscopic Xp21 duplications are thought to be healthy and fertile due to skewed X-inactivation, preferentially inactivating the duplicated chromosome and thereby protecting the individual from increased gene expression. Similarly, a skewed inactivation in carriers of the small duplications would indicate a deleterious effect on increased *NR0B1* (*DAX1*) gene expression on ovarian function.

## 2. Materials and Methods

### 2.1. Patients

**Figure 1:**
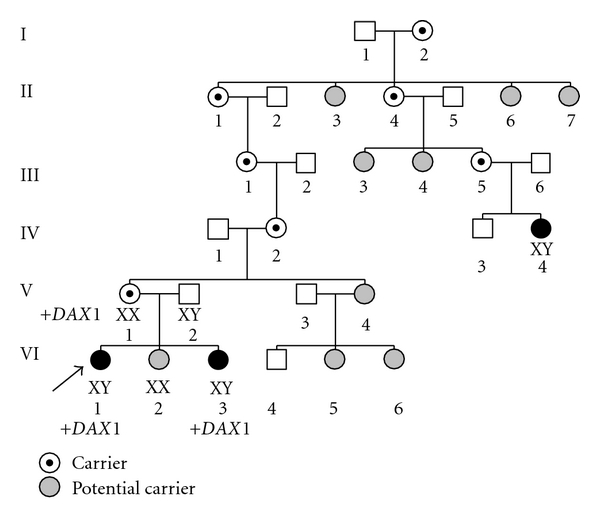
Family pedigree. Pedigree of a family with two sisters with 46,XY gonadal dysgenesis. +DAX1 indicates the subjects that carry the X chromosome containing the *NR0B1* locus duplication. XX and XY have been added underneath some subjects indicating a 46,XX or 46,XY karyotype, respectively. Mandatory female carriers and potential carriers are marked.

We studied an English family (Figure 1) with two sisters with isolated 46,XY GD. An X-linked inheritance of the defect was suspected on the basis that, through the maternal line, there is a relative who is an XY female. Informed consent and ethical approval were obtained.

*Patient VI.1 *(*proband*) presented with primary amenorrhea at 16 years of age. She had female external genitalia. The endocrine profile showed markedly elevated gonadotropins and low oestradiol. The testosterone level was 1 nmol/L and androstenedione was 2.4 nmol/L. Chromosomal examination and revealed a 46,XY karyotype. At ultrasound examination and the vagina was normal with a small prepubertal uterus of 1.8 mL of volume. A small amount of tissue of 0.5 mL volume was visible in the region of the right gonad, no follicles were identified within it. No ovarian tissue was identified on the leftside; however, this may be due to technical problem rather than the gonad being absent. At 17 years and 5 months of age, she underwent gonadectomy. Histological examination revealed fallopian tubes, and streak gonads. Ovarian-type stroma could be detected, and in one area some follicle cells could be demonstrated.

*Patient VI.3* is the younger sister of patient I1. She also had a 46,XY karyotype. Ultrasound examination, at 12 years and 4 months of age, revealed a prepubertal uterus, the right gonad had a 0.2 mL volume, while no left gonad was seen. Histological examination after gonadectomy at 12 years and 11 months of age showed the presence of unremarkable fallopian tubes, ovarian stroma cells, and gonadoblastoma, bilaterally.

*Subject VI.2* has a 46,XX karyotype, she is an adult, and at the moment she does not wish to know if she is a carrier or not. After genetic counselling, no genetic investigation has been performed for this subject.

*Patient VI.4* is a female with a 46,XY karyotype; however, no more information is available. No genetic investigation was performed as DNA was not available. It is very likely that she carries the same genetic defect as patient I1 and I3.

### 2.2. DNA

DNA was extracted from blood and/or Epstein-Barr virus-immortalized cell lines by phenol/chloroform extraction.

### 2.3. MLPA Analysis

For the initial MLPA analysis, the in-house designed synthetic probe set DSD together with the EK1 reagent kit from MRC Holland (Amsterdam, The Nederland) was used as previously described [10]. Several probe mixes (Table 1) with different combinations of probes targeting sequences within the Xp21 region were used to further narrow down the breakpoint regions.

### 2.4. Fluorescence In Situ Hybridization

Fluorescence in situ hybridization (FISH) analysis on metaphase nuclei prepared from EBV-immortalized lymphocytes was performed as previously described [9], using the BAC clone RP11-662D2 (CHORI BACPAC Resource Center, Oakland, CA, USA) which contains the *NR0B1* gene.

### 2.5. X-Inactivation Experiments

The X-inactivation pattern was examined by the methylation analysis of the polymorphic CAG repeat within the androgen receptor gene [13]. Briefly, 250 ng of DNA were digested using the methylation sensitive enzyme HpaI; digested and undigested DNA samples were amplified by PCR using a primer pair flanking the CAG repeat region as well the cleavage site, with the forward primer FAM labelled, Taq Gold (Applied Biosystems), 1X buffer II (Applied Biosystems), 200 *μ*M of each dNTPs, and 1.5 mM MgCl_2_. PCR products, together with the ROX HD400 size marker (Applied Biosystems), were size separated by capillary electrophoresis using a ABI3100 genetic analyser (Applied Biosystems, Warrington,UK). Trace data were analysed using the GeneMapper software (Applied Biosystems). Peak heights for the two digested alleles were corrected by the peak heights of the corresponding undigested alleles. The ratios of the skewed X-inactivation in digested samples were calculated by normalizing the sum of the two corrected alleles to 100%. DNA was obtained from EBV-immortalized cell line and from blood. In addition, DNA from a male sample was included as control for complete digestion. Data are presented as the average of three independent experiments.

## 3. Results

### 3.1. MLPA Detection and Fine Mapping of the Duplication

**Figure 2:**
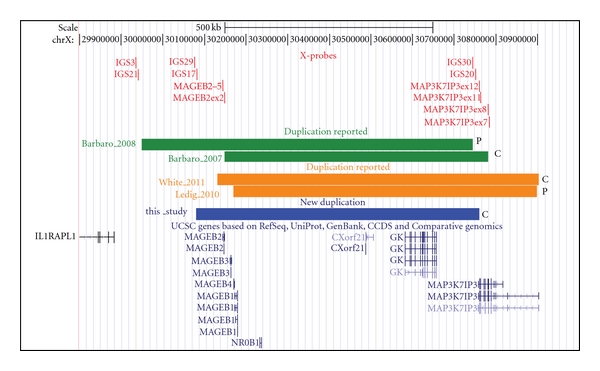
Comparison of *NR0B1* locus duplications. Representation from the UCSC genome browser (NCBI36/hg18) of the *NR0B1* locus on Xp21.2. The probes that delineate the breakpoints in the three independent families identified by our group are represented by red vertical lines. The horizontal blue line represents the extension of the duplication in the family here described, the green lines represent the duplicated regions previously described by our group, and the orange lines represent the two duplications identified by array CGH in patients with isolate 46,XY GD by other groups. The C and P at the right ends of the lines indicate if the GD was complete or partial, respectively.

The MLPA analysis with the DSD probe set detected a duplication of the *DAX1* probe as well of the MAGEB1last probe, targeting the last exon of the* MAGEB1* gene, in both affected sisters. MLPA was also used to further characterize the breakpoint region, using several probe mixes with different combination of probes targeting the Xp21 locus. We determined that the telomeric breakpoint region (5.6 kb) is located approximately 63 kb upstream of the *MAGEB* genes, while the centromeric breakpoint region (2.6 kb) lies within intron 11 of the *MAP3K7IP3* gene. Thus, the duplication has a minimal and maximal size of 679 kb and 687 kb, respectively, and in addition to *NR0B1* it contains the *MAGEB* genes, *CXorf21*, *GK* and part of the 3′ region of the *MAP3K7IP3* gene (Figure 2).

Parental DNA analysis revealed that the mother is a healthy carrier of the duplication. Table 1 summarizes all the probe pairs used and the results obtained. Together with these English patients, we present, for comparison, the results obtained for two Iranian sisters [9] and for an Italian patient, in which we previously narrowed down the breakpoint region [10].

### 3.2. Analysis of the Duplication Using FISH

Metaphase FISH analysis was performed to establish the location of the extra copy. On metaphases from the patient, only one signal for the clone RP11-662D2 containing the *NR0B1* gene was detected; on metaphases from the mother only one signal per X chromosome was detected, thus indicating an interstitial duplication and excluding translocation of X chromosome material onto another chromosome (data not shown).

### 3.3. X-Inactivation Studies

We analysed the X-inactivation pattern in subject V-1, and the methylation ratio between the X-chromosome carrying the duplication and the normal chromosome, in EBV-immortalized lymphocytes, was 65% : 35%. We performed X-inactivation studies also on two other mothers carrying different *NR0B1 *locus duplication that have been previously described by our group [9, 10]. Ratios between the X-chromosome carrying the duplication and the normal chromosome were 58% : 42% and 33% : 67%, respectively.

These results indicate that in lymphocytes there is not a strong preferential methylation for the X-chromosome carrying the duplication.

## 4. Discussion

We present here a family where a relatively small *NR0B1* locus duplication is the genetic cause of isolated complete 46,XY GD. The duplication extends from the *MAGEB* gene to part of the *MAP3K7IP3 *gene, including *NR0B1*, *CXorf21*, and *GK* genes.

By bioinformatics evaluation of the breakpoint regions, we noted that within the telomeric breakpoint region there is a *AluSc* repeat of approximately 300 bp that shares 85% and 84% identity (BLASTN2.2.21 [14]) with two *AluY* repeats within intron 11 of the *MAP3K7IP3 *gene. These evolutionary young *Alu* repeats may be involved in the duplication mechanism as they are found to be enriched near or within duplication junction [15]. We unfortunately have not been able to amplify and sequence the duplication junction so we could not determine the rearrangement mechanism and differentiate between a nonallelic homologous recombination (NAHR) or a nonhomologous end joining (NHEJ) mechanism. The latter was the mechanism of the duplication in a previously described family [9]. Actually, the failure, after several attempts, to amplify the duplication junction in two of the three duplications we have identified, makes us suspect that the duplications could be more complex and be generated by a FoSTes (Fork Stalling and Template Switching).

Xp21 duplications, as well deletions [16], including the *NR0B1* gene, are all different, thus they are caused by nonrecurrent rearrangements. This indicates that in this region there are several genomic elements that can lead to genomic rearrangements. These rearrangements will be different regarding size and genes involved. This is important to consider when a genetic test is chosen or developed to screen for these genomic disorders.

Two other groups have applied array-CGH to analyse patients with 46,XY gonadal dysgenesis and found additional Xp21 duplications [11, 12]. Patients with large duplications presented XY gonadal dysgenesis associated to syndromic feature, while two patients with relatively smaller duplications (<1 Mb) including *NR0B1* presented isolated 46,XY gonadal dysgenesis (Figure 2). Thus, confirming our findings that *NR0B1* locus duplication should be investigated in all cases of isolated 46,XY gonadal dysgenesis.

By comparing the three small Xp21.2 duplications identified by our group, together with the other two recently reported by Ledig et al. and White et al. [11, 12] (Figure 2), it is not possible to directly delineate a genotype-phenotype correlation for the partial or complete GD forms. It is known that dosage-sensitive genes with complex expression patterns are particularly sensitive to positional effects, and regulatory regions can lie far outside the transcription unit [17]. A regulatory region upstream *NR0B1* has been proposed after the identification of a 257 kb deletion in the region between *NR0B1* and *GK* gene in a patient with XY GD [18]. Furthermore, a small inversion immediately upstream of *NR0B1* has been identified in a patient with congenital adrenal hypoplasia [19]. However, patients with both partial and complete form share entirely these regions in their duplications. The interaction of *NR0B1* with other transcription factors could also modulate the final phenotype.

Interestingly, in all cases with isolated 46,XY GD, the *IL1RAPL1* gene, located immediately telomeric to the duplication containing *NR0B1*, is not disrupted (Figure 2). Deletions or mutations of this gene have been identified in patients with mental retardation [20]. Disruption of this gene could explain the mental retardation in some previously described patients with larger Xp21 duplications.

As all three duplications we identified were maternally inherited, we could use the material available to investigate the X-inactivation pattern of the duplicated X. In several studies where a larger Xp21 duplication was maternally inherited, it was shown that the duplicated X was preferentially inactivated [21, 22]. Thus carrier women are thought to be protected from a double *NR0B1* dose on the gene expression level. Therefore, we investigated if there was a preferential methylation of the duplicated X chromosome also in females carrying the small duplications which contain fewer deleterious genes. Analysis in lymphocytes of the three mothers showed that there is no obvious preferential inactivation of the duplicated X. Even though we could not investigate the situation directly in the ovary, we can hypothesize that healthy carrier women are fertile because the ovary, in contrast to the testis, can tolerate an extra dose of *NR0B1*.

In conclusion, the identification of *NR0B1* locus duplications in patients/families, originating from different countries (Iran [9], Italy [10], England (this study), Germany [11], and Australia [12] stresses the importance of using methods that can detect submicroscopic duplications of the region surrounding *NR0B1* in the evaluation of patients with isolated 46,XY GD (complete or partial). The lack of phenotype in the carrier mothers and the pedigree of the family here described also illustrate that such duplications can be spread through the female line in the family. In the present family there are seven mandatory female carriers and nine potential carriers. In fact, some of these could even be affected XY females who have not been investigated. Small duplications are most probably more frequent, but have escaped detection due to the methods that have been used so far and the selection of the patients investigated. We believe that in a patient with isolated XY GD, the *NR0B1* locus should be carefully investigated. MLPA and array-CGH are two different techniques that can be applied. Array-CGH offers the advantage of a whole genome screening approach; however, the capacity to detect very small *NR0B1* duplications depends on the platform used (number and distance of probes within the *NR0B1* locus). *NR0B1* has a size of 5 kb, and the two nearest *AluSx* sequences on opposite side of *NR0B1* are only 14 kb apart. MLPA containing specific probes guarantees the identification, of isolated *NR0B1* duplications and the maternal sample can be simultaneously analysed for carrier status at a limited extra cost compared to array CGH.

## Figures and Tables

**Table 1:** MLPA probes and results.

Probe name^a^	Size (bp)	Results^b^			Sequence^c^	
		This study	[9]	[10]	5′ half probe	3′ half probe
IRLAPL1	126	−	−	−	CAGCTCTAGCCACTGCCCATCCAGATCTCCGTTCTACCTTTCACA	ACACGTACCATTCACAAATGCGTCAGAAACACTACTACG
IGS10	93	−	−	−	CTATGCTGCCAGAAGTCTTAGCAA	AATGCCACATGTGCCCAAGAGAGATTC
IGS11	90	−	−	−	GGAAAATCTCTCTGGGCAGCTGCA	GCCACAGTGCAGGTTGCTGACTTC
IGS15	117	−	−	−	CACAATTTGGAGCAACTTAAAGAAGATATCAGAAATT	TTCTCCAAATTCTGTAGAAAGCACAAATTAAAACAGAG
IGS16	102	−	−	−	GTTTTCTAGAGCCATGTATGAGATTGCAGA	TGTGAATATTGTTCCAGTGAGAGGATACTG
IGS28	99	−	−	−	CTAAATGCTAAAGTAATGGTGCCAGTAA	AAGTGACATTGGTAACTTTGGAAACTCTG
IGS29	93	−	−	−	GCACTGCGGCCTTCTGACATCAGGTATG	ACTCCCTGCCCTGATCTTTCTAC
IGS17	114	−	−	+	GAGTAGATCTCCTTACAGAGAAGCTAGTTGTGGATG	TTGTCCCCTTCTTCTTCCTGAATGCATTCCTTCAGG
IGS12	111	−	−	+	GAAGGAATTCTAATATCAGCAGTCTGTGAACA	ATGTGAAAATCACTGCCTAAGTCTATTGCAGGCACAG
IGS13	117	−	−	+	GCTTCCCCCTTGGATTCTTTATAGCCAATGCACTTA	AAATAGGTTGTTTTGGCACAGCCTGCACAGTTTGATACC
IGS1	99	−	−	+	GGAGGAAAGTAGGACAATTTGGAGGA	ATTTGGGTTCTCAGTGGGGTGGCATTGTAGG
IGS2	117	−	−	+	GTGTATGATAACTGCGTGTCCTCACTGTGAATAATAGCTTG	TGGAACACAAACTGATAGAAATAGACGCATATTG
IGS3	111	−	−	+	CAGCAGCTGGTGAGTGTTCAGCATTAGATATGAGAAGTTA	AGCTGAATAAACTGGAGGAAAACCTTCTG
IGS21	108	+	−	+	GTATCAATGATTTGGGGTTCTGTGTTC	TATAAGCTACAAGACCTGCCCACCACATATTTACACTTC
IGS22	90	+	−	+	CCCAATTCCTCAGTTACTCACTGG	TAGTAAGCTGGACCCTTAGGGAGG
IGS23	126	+	−	+	GAGCATATTTACATGCACCTGGAGGAAGTATCTGATACTATTT	CTAAAGATTAAGTAACAGTGCCTCTTCACTCTTGAATCTAG
IGS24	120	+	−	+	CTTATCCAACTAGGCCTTACATAACAAGTAATAGTCTCCAG	ATGGAATGGCAAAACAACTCTACCAGTGCACTACATC
IGS26	93	+	−	+	GATTAGACAATGTGGGCAGCTATCT	TGGCAACATGACATAATTGGAGCTGC
IGS6	117	+	−	+	GTGGCATAAGGAGCTCACAGCTAGAGTTACT	ATCAAAAGCTAGGCAAGCAGAGAAATATTTATGCCACTATAGCC
IGS7	90	+	−	+	CTGCTAGATGAGCGCACCTCA	ACAGCAAGAGGCTATAGGATGTTCTGC
IGS8	102	+	−	+	CATCCTACATTAGATGATCTCAGTCAACCA	CTGACAAAGACTGAAGTTCAAATTCTGATG
IGS9	108	+	−	+	CCCTCAGATGGTAAGCTTCAGAGTTGAGAGACT	AAGCCTATAATGGAAAAGTGCTCTTAACTTTGG
MAGEB2-5′	96	+	−	+	GGACTGGCGGATTTGGGTCAGCACGCAT	ATTCGTCCCAGGCTGCTAGATACTGC
MAGEB2ex2	93	+	+	+	GGCCACACTTACACCTTCATCGACA	AGGTAGACCTCACTGATGAGGAATCC
MAGEB1	120	+	+	+	GAAGGAAGACAACCCTAGTGGCCACACCTAC	ACCCTCGTCAGTAAGCTAAACCTCACCAATGATGGAAACCTGAGCAG
MAGEB1last	113	+	+	+	GCTAAACCTCACCAATGATGGAAACCTGAGCAATGA	TTGGGACTTTCCCAGGAATGGGCTTCTGATGCCTCTCCTGG
DAX1	102	+	+	+	GCAGCCTCAGCGGGCCTGTTGAAGACGCTG	CGCTTCGTCAAGTACTTGCCCTGCTTCCAG
IGS4	132	+	+	+	CCAAGGAAGTCATAACAGCACAATAGCTATAC	AATCTGATAACCTTGTTAGCTCAAATCAAAGCTCCTAACAAGTAGGAGAGGTTAGTGC
CXorf21	90	+	+	+	CCAGCCACCTGCTCATTATAAGAG	GCACAACTCCAGTGGATGTCATTC
GK	96	+	+	+	CACTATATGTAGGTTATCTCTTCGGTGACA	TACACTGCAATTTGAGAGGGCTGG
IGS5	114	+	+	+	GCAAGCAAGTAAGTGCTGTATCTCCTAGCGAC	TCCCTCCATCACCATTCCTGGTACTCTGTTGTCAAATGGC
IGS14	96	+	+	+	GGCAGAGTCCAATCTATAGCAGAGGAA	*t * CAGCAGCAGTAGAGAGAGTATAAAGC
IGS18	99	+	+	+	GAGCTGGTGGAGAAACAGTTACAGAAGAT	AATGGAAAGCATGGGATCCTGGGAGTG*c *
IGS19	135	+	+	+	CCGATACTTGCATGTTGAGATGCCATCCACATTTAGCCATTTAATAC	ATAATATTGGTTTAACATTTTATCGAGGAAAGCCTGCCCTAGCACC
IGS33	96	+	+	+	GGTACTTGGGCCTGGGAAGACTGCAG	AATGGGGTTCCTGCATGCAGGCTTCGCC
IGS32	120	+	+	+	GTTTCTGTGGCAGAGATAGGCATCTTCCATTCCATTGCTGG	AGATATGTGGGGCTCCCTCAGAGAAGACTTAGAGACC
IGS31	117	+	+	+	CATCTGCCTGACATTTAACAAAGCGCATAATGGTTAGC	TGATCAATCTGGAGTCCCCACAACTTCATATATCCTC
IGS30	111	+	+	+	CAGATGTGGATATGCTGGTTTCCAATAGTAAA	CATGAATTGCTTTCAGGATTCACATTTAGGTACAAAG
IGS20	123	+	+	−	GATACATGTGACTATGGGTGATTACCTGGCATGTTTG	AGCTAAGCCTTTATCACATAATTCACATTTGTAAGGCTCCTCAC
MAP3K7IP3ex12	108	+	+	−	GCTTCTGGTAGTGCTCAAAGTTTCACTTTCAA	CCTGGCGCAGACTTTTCTGAATTCAGGTGTACCG
MAP3K7IP3ex11	123	−	+	−	GTGTCATGGATGTCTGCCTGTACTTTGGAGGTCACGCTA	ATTCTTCGGGCTTTTCTCTCAATTGTGCAGGGGTCTGAGGAG
TAB3ex10	102	−	+	−	ATGGCTTTTGGATCAAAGTTTCCTATAAA	GAAAGTAAATTCATCAGGCATTTAAGAACTC
IGS27	99	−	+	−	GTAATTAGAACAGGAAGAATGGGGGGGAAT	AACTTGAGGGAGGGGGTAATTGGCAGC
MAP3K7IP3ex8	99	−	+	−	GGGATCGCAGTGGTGCAGCTGACTCTTC	TGAGCCGTCTCTGCATCAGGTCATGCTCC
MAP3K7IP3ex7	111	−	−	−	CGGCAACAGGCTTCAAGATTGTTGTTATTC	TAGGGGAGAAAAATGGTAAAAGTAACATTGGCAACATTC
MAP3K7IP3ex6	114	−	−	−	GTGGCCAAGTTTCTCTTAGGAAATGGATGTT	AACCGGCTTTCCAAAAGTAATGATCTTCTAGCACCACAGTC
MAP3K7IP3ex1	123	−	−	−	GCTTGGACTCTGAGGTTCTGACCGTAGCATCAGATCACACAGAGAAC	TACCTTGCTCGCCCGAATGTGTGGCTTTCTCTGC
DMD	135	−	−	−	CATCGCTCTGCCCAAATCATCTGCCATGT	GGAAAAGACTTCCTACATTGTGTCCTGGAAAACAAAGAGAAAGAAAGACAGACTTTACAAAAGG

^a^The probes are listed according to their genetic location from the most telomeric to the most centromeric.

^b^Plus and minus signs indicate duplicated and nonduplicated regions, respectively.

^c^The 5′ half probes are preceded by the universal tag sequence GGGTTCCCTAAGGGTTGGA; the 3′ half-probes are followed by the universal tag sequence TCTAGATTGGATCTTGCTGGCAC and are phosphorylated at the 5′ end.
